# Charisma: The phenomenon and its psychology: A mental health perspective

**DOI:** 10.4103/0019-5545.44751

**Published:** 2008

**Authors:** Ajit V. Bhide

**Affiliations:** Psychiatrist, Bangalore, India

(This paper is based on the Dr. LGP Achar Memorial Oration delivered by the author to the Indian Psychiatric Society, Karnataka State Branch, at its Annual Conference at Manipal on 30 Aug 2008)

At the outset, I thank our society for the honor bestowed on me today by asking me to deliver the late Dr. LGP Achar Memorial Oration. Dr. Achar swept into the activities of our society; like a whirlwind. Thanks to his efforts, our membership swelled. His was a visible presence in most of the society's activities, outdoing both youth and veterans of the time.

It is with respect for his spirit and his devotion to psychotherapy that I humbly deliver this talk.

## CHARISMA, TRANSFORMATION AND PSYCHOPATHOLOGY

There is in some individuals the remarkable ability to affect other persons, communities, and even populations, in such a way that dramatic transformations are effected. This ability is sometimes consciously exercised and much less commonly, it is totally unwittingly practiced. The results too vary, between those brought about by design and those brought about totally by happenstance. Then again, the design might be altruistic or sinister.

In today's presentation, I intend to focus on this uncommon ability and touch upon its relationship to the field of mental health. Charisma is a general term bequeathed to us by the German sociologist Max Weber (though not coined by him), who studied the phenomenon assiduously. Charisma has had its maximum effect in religion, politics, and the arts. The profound and often ineffable effects of charisma on the ways of thinking are a challenging subject to study. So are its antecedents.

### According to Weber, charisma is:

*“…a certain quality of an individual personality, by virtue of which s/he is “set apart” from ordinary people and treated as endowed with supernatural, superhuman, or at least specifically exceptional powers or qualities. These as such are not accessible to the ordinary person, but are regarded as divine in origin or as exemplary, and on the basis of them the individual concerned is treated as a leader.”*[1]

Writer and scholar David Aberbach, a keen student of charisma, observed that the exploration of secular charismatics about whom much is known (such as Hitler, Thatcher, Chaplin, Dev Anand or Natasarvabhouma Dr. Rajkumar), casts light on the nature of charisma in its religious forms where little is known. Conversely, the study of religious charismatics often helps to interpret what seem to be exclusively secular phenomena. Secular charismatic phenomena can be exemplified by Subhash Chandra Bose's role in India's freedom struggle or that of Maximilien Robespierre in the French revolution, or those of Giuseppe Mazzini and Giuseppe Garibaldi in the Italian struggle for independence. Gandhi, essentially a philosopher politician, with his strong religious streak and moral rectitude, presents the true overlap between the religious and the secular charismatic.[2]

While popularity is a vital ingredient of charisma, it is not its exact equivalent. Shakespeare was tremendously popular for his writing. Recorded history accords him no suggestion of charisma.

One recent writer on the subject, Philip Rieff, believes that charisma lately ‘has been battered to death’.[3]

## THE ORIGINS OF CHARISMA

Psychoanalytic literature often traces charisma as originating from a variation of the manifest need to relate, that an infant innately has. If the infant's needs are well met by its caregivers, then the appeal for relating sublimates into quiet confidence and affection, mainly towards loved ones. Thwarted, this appeal may go on to promiscuous and even pernicious exaggeration: an over-blown drive for the mastery denied to the small one. In adult life, the charismatic may continue to reach towards the infinite, to merge with a greater self. This may take the shape of seeking merger with society or humanity at large, through the mastery of a medium such as religion, politics, literature or other art forms. When the search is accompanied by natural gifts in the medium, the recipe for charisma is even more successful.

Parental deprivation through death, separation or ineffectiveness often renders the child or adolescent insecure. Emotional handicap or disability is sought to be compensated by a quality of attractiveness. The charismatic's gifts come to fruition through an intersection between his or her inner world and external social reality that continuously impinges on the individual and the collective psyche. The relationship between the charismatic and the group is innately paradoxical. Weber observed that charisma emerges and exists in relationship to a group, especially in crisis. It is reminiscent of the verse in the Bhagavad-Gita when Sri Krishna declares that the divine incarnation will be manifest whenever dharma (righteous life) is endangered.

Crises brought to the fore several charismatic leaders even in recent history and thrust them forward. However, sometimes, the individuals themselves created crisis. This was done to resolve inner inadequacies on a public platform, often unmindful of the consequences to the greater lot. The motives and needs of the charismatic and the group could be widely disparate with the narrowest of intersections.

Not all charismatics have their unique charisma originating in childhood privation or trauma, but those who do not, seem significantly fewer. The privileged prince Siddhartha Gotama was nothing more until in adulthood he witnessed what had with great artifice been hidden from him by his well-meaning father: the reality of human tragedies of sickness, penury, aging, and death. The sad discovery drove him to despair and to search for a meaning in life that he finally claimed to find after prolonged ascetic practice, and charisma was not far behind.

The attainment of charisma comparatively late in life is a well-known phenomenon, though the exception rather than the rule. Most charismatics attain their peak of appeal in their thirties and very many continue to draw their followers for decades thereafter.

Interestingly, though not necessarily frequently, charisma begets charisma. I do not speak here of the political mantle and the aura that goes with it, that have become hand-me-downs in many so called democracies including ours, but of true inspiration that derives from one Master to another. This may happen in the actual proximity of the two, as in the case of Ramakrishna Paramahamsa whose divine grace, unending devotion, and unfathomable love converted the young and initially skeptical Narendranath Dutta to become filled with discernment (Vivek) and bliss (Ananda) and fired in him a passion to become a charismatic with few parallels. The inspiration may come from a distance, as in Vinayak Damodar Savarkar's from Mazzini, whose biography shaped the young Indian patriot's zealous chauvinism.[4] Gandhi was galvanized by both, the stories and the life story of Leo Tolstoy, a charismatic himself, who lived by his chosen principles in defiance of orthodoxy in a once great country that had become decadent.[5]

## PHILOSOPHY, RELIGION AND CHARISMA

The centuries from 800 BCE to 300BCE saw the burgeoning of philosophical and religious concepts that was unprecedented. Jeremiah, Jewish prophet and scholar, Gotama Buddha, the founder of a whole new religion, Mahavira, the chief preceptor of what became a rival religion in close proximity to Buddha's, Lao Tzu, a central figure in Taoism, Confucius, an independent thinker also in China, flourished in this time. In the West, ‘the ugly of face but sharp and steady of mind’, Socrates, wielded his influence at the close of this period and left an indelible imprint on the way Occidental man thinks and behaves to this day, emphasizing the spirit of personal inquiry. All these thinker leaders were effective principally because of their charisma. Personal scars may have propelled them to this achievement on the background of a milieu of uncertain values and a thirst for better reasoned norms.[6]

Weber spoke of charisma ranging from the ritual ecstasy of primitive religion, to the euphoria purveyed by the ethical prophet to the political passion stirred up by the charismatic union of leader and follower. Many religious leaders wield their influence over their followers long after they are gone.[1]

Charismatic religious leadership is often infused with political strategy. Moses, apart from getting Divine Law to his people, united quarrelsome slave bands to a promised land and made them a fighting nation. Jesus not only taught the principles of faith, hope and charity; he lethally challenged the imperial status of Rome by declaring God to be the only true monarch and the eternal kingdom of Heaven to reside in the hearts of the faithful. Mohammed did not only start a new religion; as an astute military commander he conquered the Arabic lands and united barbaric tribes and gave at Mecca a center to their nomadic life, preparing the way for Islamic expansion.[2]

Each of the major religions of the world, including those that today have a reduced populace such as Zoroastrianism, and with the noteworthy exception of Hinduism, has a solitary charismatic prime mover. Doctrinaire rigidity which was often never intended by the progenitor serves to dilute the effectiveness of this religion; this might well be considered a dangerous adverse effect of religious charisma.

A parallel in our times of great relevance to us is the non-eclectic passionate adherence of hardcore Freudians, Adlerians, and Jungians to the originators of their ‘schools’. Marxists, Darwinians, and acolytes of many other original path breaking thinkers, all have a code of allegiance not unlike Pentecostals and other rigid religious sects.

In our own country, there was probably no greater charismatic religious leader than Adi Shankara, who sought sanyasa at a very tender age, traversed the country with fervor, looking for enlightenment. After his initiation by a Guru on the banks of the Narmada, he pursued the spread of Advaita philosophy with truly missionary zeal. His charisma was strengthened by his quick-witted linguistic and poetic skills, firmness of faith, and argumentative excellence.[7]

Many are the charismatic saints of India: The twelve Alzhwars of Tamil tradition, literally ‘immersed’ in their devotion, most notably Nammalvar, through their lyrical discourses enthralled and sparked devotion in millions. The Periyapuranam describes the lives of over 60 ancient Shaivite saints of the Tamil lands, many of them profoundly charismatic.

This became the first source of inspiration for a rather unusual saint of our times, Ramana Mahrshi. Ramana was uncommon, for his charisma like his ascetic practice, was primarily silent! His silence much more than his discourses stilled thousands of troubled minds and converted the doubting visitor, novelist W. Somerset Maugham, to believing in spiritual salvation.

Basava's iconoclasm in fighting sectarianism and meaningless ritual, as well as his prudence, gained him a mammoth following. The Bhagvat tradition of Maharashtra produced the charismatic Dnyaneshwar, Namdev, Eknath, and Tukaram. Madhavacharya, Purandara Dasa, Kanaka Dasa, Raghavendraswamy, are other magnetic religious leaders of this land, their charisma reinforced in some instances by a talent for music. Three others, Ramanujacharya of southern Mysore region and Chaitanya Mahaprabhu of the Orissa/Bengal tradition and much later, Narayana Guru of Kerala, are recorded as being charismatic enchanters.

In more recent times there have been many claimant avatars and gurus but a few like Ramana have never sought to draw crowds, but have never turned a seeker, whether of immediate materialistic relief, or of long-term salvation, away. The mendicant Sai Baba of Shirdi, shunned luxury and in many ways emulated his role model, the charismatic Sant Kabir. In contrast, many swamis and gurus of today profess a notable charisma but cater largely to a privileged clientele, basking in the luxury of palaces of marble and glass, limousines and velvet covered thrones. Some became obsessive collectors of precious stones and designer vehicles! There are hugging saints and unwinding saints, literally breath holding gurus, saviors who are joggers and motorcyclists, each with an impressive following.

Mother Theresa lived the life of a true saint, though to this day she has her detractors. Her piety, as much as her courageous bonding with the diseased and downtrodden, her unpretentious commonsense advice to those who sought it, rendered her a unique place in the hearts of millions. Her story reminds one of a great charismatic, St Damien, patron saint of leprosy, who worked tirelessly among sufferers of this disease. He used to say ‘we lepers’ even when he was not one, and was actually thrilled when he did acquire the condition, as now his identification with his followers was thorough.

The term charisma itself was largely restricted to the ability to perform miracles by divine intervention, among Christians, especially Roman Catholics, till Weber widened its scope.

Most saints are charismatic pacifists. More militant saints who were religious zealots, politically driven by a sense of justice, or more appropriately, a need to undo injustice, include the charismatic Ramdas Swami, Shivaji's guru, and the ruthless devout charmer, wielding the sword to defend his faith, to stop the evil of forced conversion, Guru Govind Singh.

It is worth noting that early parental demise is a marked turning point in the lives of very many saints. Other traumata, both personal and vicarious have also been known to accentuate or even engender their turning to spirituality.

## CHARISMA IN POLITICS

Political charisma, even in democratic states that separate governance from the church, retains many features of traditional religious charisma. The background, inner life, and psychology of many political charismatics resemble those of religious leaders and saints.

There are important differences, though. Great religious leaders are believed in long after their deaths; and as Aberbach observes, when the matter of faith enters the comparison, it throws into relief the erosion of charisma in modern political life.[2] Political charismatics draw on subconscious motives and drives, using the force of their imagination to extend the possibilities of existence, at least ostensibly, for the general good of the people, their wished followers. Gauging the impact of such leaders is a daunting task.

One approach is to imagine the course of history if the opposite had happened, thereby excluding the leader at a crucial temporal stage. If Robespierre had been executed a half decade earlier in the French revolution; if Hitler's covertly conspiring officers had indeed blown him up. for that matter if Gandhi had anointed Vallabh Bhai Patel rather than Jawaharlal Nehru as India's principal leader, could history have been very different? These ‘what-ifs’ are innocuous intellectual exercises that bring into focus the irony of modern states having been shaped by unpredictable forces and by a handful of individuals.

Many of these leaders have very ordinary lives and circumstances seem to conspire to bring them to the fore: George Washington, a quiet planter was transformed into a continental commander. Mohandas Gandhi was ‘a mediocre, unimpressive, floundering Barrister-at-law’ in sharply contrast with the Mahatma, leader of millions. Public cause ‘tapped his enormous reserves of intuition, will power, energy and self-confidence’.[5] Giants like Garibaldi, Lincoln and Lenin, had pronounced ordinariness about them that would not have predicted their future greatness. Churchill achieved a charismatic bond with his people quite late in life, when circumstances brought about his ‘finest hour’.

The most grossly underestimated of modern charismatics, arguably, was Adolf Hitler. Like a prominent minister in our country, now back on the track of fortune, Hitler was once dismissed as a ridiculous clown. The main attribute that drove him to awe inspiring greatness was his fanatical racist conviction. Hitler's biographer Ian Kershaw, notes that, ‘the mass appeal of the charismatic leader has only an indifferent relation to that leader's actual personality and character attributes. Perceptions are more important than reality.’[8]

Great political charismatic leaders have an abiding conviction that destiny has chosen them for a heroic mission. Shivaji was motivated by the conviction of a divine direction to work for establishing the ‘Hindvi Rashtra’.[9] Napoleon often spoke of destiny. Garibaldi believed in his destiny to triumph, and this firm belief engendered his fearless fighting and power to inspire people.

Often the mission begins with a crisis; the charismatic leader rises from the ordinary to determinedly fulfill a destined role. Napoleon might have remained an obscure officer with a limited command but for the crisis that brought him to power. His adversary remarked that Napoleon's presence on the battleground was worth forty thousand men; but what if there had been no war?[2]

Sometimes, aspirant leaders welcome, or even create crisis. Indira Gandhi, piqued at being perceived as a puppet prime minister doing the bidding of wizened power hungry men, seized the opportunity to show them the door when the opportunity arose three years into her premiership. It was after this that the charismatic, self-assured, shrewd Indira emerged.[10]

Franklin Roosevelt, crippled by polio in 1921 at the politically young age of thirty-nine, spent his last twenty-four years without the use of his lower limbs. The spiritual battle of his affliction was at the core of his charismatic appeal as President of the USA during the depression and World War II. His record of service before his illness was not impressive and he was perceived as being, vain self-serving and arrogant.[2] Personal crisis that brought him great ignominy had dealt him a literally crippling blow and he came down with the polio paralysis.

Seldom have the effects of adversity been sweeter. Rising above the defeats of health and reputation, he fought back and was transformed into a man of vast spiritual vigor from an unethical politician. The climax was his being elected to the White House while the country was forlorn in crises. Today Roosevelt, once dismissed as corrupt and unfit, is rated as the greatest President ever of the USA. As one who had battled despair, he was righteous in declaring, ‘the only thing we have to fear is fear itself’, a slogan that wildly caught the imagination of a nation browbeaten by mediocre governance.

Margaret Thatcher, in a comparable mode almost half a century later, with her country having been for more than a decade under unremarkable leadership, embraced a simple slogan, ‘Britain has lost its way’. The erstwhile seat of the World's widest and most prosperous empire had been reduced to a second-rate power. She quoted a predecessor, ‘I know I can save this country and that no one else can.’[11] Voted to power, she lived up to the promise and in a few years Britain gained both self-esteem and the esteem of its peer nations. Criticized for her blatant capitalism and no-nonsense handling of labor precipitated crises, Thatcher remained undaunted, leading the country from strength to strength and remaining in power an unprecedented tenure of eleven years. Thatcher's charisma was widely respected but she was largely a lonely person, often the price of greatness.

In studying charismatic politicians, Fidel Castro, Adolf Hitler, Vladimir Lenin, John Kennedy, Ferdinand Marcos, Gamal Abdel Nasser, all present fascinating lives where the yearning for public approval at least in some measure springs from unrequited love, significant privations or trauma in childhood or adolescence. Equally, there are Giuseppe Garibaldi, Jawaharlal Nehru, Vallabh Bhai Patel, Sun Yat Sen, Mao Zedong in whom not personal trauma but burning idealism and an inner need for justice seem to be the motivators. Ruthlessness is an accompaniment of charisma in most political leaders, all the more so in those from totalitarian states.

Wisdom does not necessarily bestow charisma. Gandhi's political guru, Gopalkrishna Gokhale, was not charismatic though he was brilliant and sagacious. On the other hand, Bal Gangadhar Tilak, senior in age to both Gokhale and Gandhi, and ideologically their opponent within the Congress party, favoring militant nationalism, was an orthodox Brahmin, astute and acerbic writer and editor, and gained charisma across caste barriers, which at that time was unique. A worthy successor of Tilak's kind of charisma was the legendary Subhash Chandra Bose, removed prematurely from the Congress by the ruses of Gandhi, and removed from greater historical glory by fate.[12] Three courageous charismatics stand out for their contribution for an egalitarian society in this country: Mahatma Jyotiba Phule, Babasaheb Ambedkar, and Shahu Maharaj.

There has recently been a comparison of the two great political charismatics: Gandhi and Churchill.[13] Winston Churchill, virtually abandoned by his aristocrat parents in infancy, was raised by his nanny who remained his only close friend and confidant in his early years. His poor behavior at boarding school may have resulted from the misery of his abandonment of sorts, and the failure of his parents to respond to the young lad's repeated pleas to visit him at school.

Psychotherapist Anthony Storr records that Churchill was exceptionally prone to bouts of depression, that he labeled his ‘Black Dog’. Belligerent and hostile, Winston was much admired for his linguistic, military and strategic skills, even his cussed bravery, but liked he was not.[14] His charismatic appeal came to the fore in 1940 when the nation, beleaguered by a sinister war against Nazi Germany desperately needed a bellicose articulate leader to match Hitler's wily ways, and the aging ‘Bulldog Warrior’ fitted the bill. There were other facets of his personal life that had shaped young Winston's sense of both frustration and defiance and finally the latter came to his rescue.

Mohandas Gandhi was a determined fighter who was also determinedly weaponless in the military sense. Armed with moral righteousness and seeking but equal respect for all humans, he resorted to the enforcement of truth as a stratagem. His style has been likened to a passive aggressive mode with some justification, and his moral snobbery reinforced by his almost boundless charisma, has even rendered him a ‘comissar’[15] in the eyes of some. In his early fights, Gandhi showed the spirit of Buddha and Christ could be applied in modern times. ‘He seldom preached about God and religion’, wrote his biographer Fischer, ‘he was a living sermon.’[5]

Messianic stances of political leaders do render them quasi-religious. This can sometimes be at a grossly physical level. The Russian priest Rasputin, and the Italian Garibaldi may have sought to resemble Jesus Christ physiognomically, consciously or otherwise.

Interestingly, Savarkar and his follower Nathuram Godse, ideological opponents of the Mahatma, enjoy a charismatic following even today that we hardly can speak of, which I personally witnessed very recently. Nelson Mandela, and Martin Luther King Jr. took great inspiration from Gandhi's methods. Each became a unique charismatic leader in his own right, not entirely due to this influence of course.

There is a phenomenon of the passing of the power baton to chosen family heirs in politics. In a culture of sycophancy and ingratiation, it is common to witness even the charisma being bequeathed to the successors. In India, the story of the Nehru-Gandhis is well known and a continuing saga. Benazir Bhutto inherited her father's legacy with charisma and aplomb. In the Phillipines, the widows of Ferdinand Marcos and his sore opponent, the assassinated Benito Aquino, could not hold on to power, clearly shorn of charisma. Martyrdom in politics, as in other fields, seems to enhance the lingering charisma of the deceased.

## CHARISMA AND THE MEDIA

The effects of modern media, especially the electronic, and now the digitalized variety have totally enlarged the possibilities and meanings of charisma. As soon as a medium makes its appearance, it is adapted to become a conveyor of charisma.[2]

In the late 1920s, radio and the newsreel, with commercial air travel- all new developments- were used with diabolical effectiveness by Hitler in his election campaign. The effect of television reached a new zenith in the 1960s, the first prominent beneficiaries probably being John Kennedy and Charles de Gaulle.[2] Handsome and articulate Kennedy's televised debates with the grossly non-charismatic but intellectually sharper Richard Nixon swung the election to the former.

Though the electronic media are clearly swifter and more easily penetrative, the effect of less sophisticated media should never be underestimated. Charismatic speakers can turn audiences; such was the gift of Gandhi, and even more so of the now often forgotten Savarkar. Justice Khosla who presided over Gandhi's murder trial commented on Nathuram Godse's concluding defense which was a powerhouse of oratory and could have overturned a whole newly liberated country, had it been allowed broadcast.[4]

Harriet Beecher Stowe's ‘Uncle Tom's Cabin’ is the novel that is said to have done more to turn the American tide against slavery in the nineteenth century than many impressive speeches including Lincoln's famous Gettysburg address. It bestowed on the authoress a charisma that long endured.[16]

## CHARISMA IN THE ARTS

Outstanding charismatic artistes share many characteristics of popular religious and political leaders, sometimes to an exaggerated degree.

There is a paradoxical co-existence of weakness and strength, apart from the creation of a new identity, and a union with a mass audience. The weakness of charismatics often springs from early family loss or deprivation, leading to lowered self-esteem, despondency and sometimes a blockage of feeling. Aberbach[2] cites the lives of Chaplin, Marilyn Monroe, and John Lennon to support these observations.

The weakness is not allowed to predominate. It gives rise to a restless craving for some unusual strength. This strength flowers in positive conditions following a trauma, and is enhanced by natural gifts which seek a creative outlet. In the struggle to overcome or master his weakness, the charismatic artist uses the media to recreate himself, to augment his worth in his own eyes, and in the eyes of his society, his constituency as it were. Marilyn Monroe basking in the adulation of a few millions, escaped the torture of her early abandonment and abuse declaring herself to belong to ‘the ocean… the sky… the whole world!’ She was adored, at best, by one-half of the human race, for her prettiness and her sexuality on screen and nothing more, but she was not going to face such a sordid fact head on.

With charisma comes the risk of narcissism, and this is maximally demonstrated among folks of showbiz.

Film stars more than anybody else end up parodying Marshall McLuhan's edict ‘the medium is the message’. On a trip to Salem not too long ago, aware as I was of the star Rajanikant's larger than life image, I was astounded to find this icon's picture adorning just about every billboard and hoarding from slick garment boutiques to hairdressers and even restaurants. M. G. Ramachandran, Rajkumar, Raj Kapoor, N. T. Rama Rao, A. Nageswar Rao, Shivaji Ganesan, Dilip Kumar, Gemini Ganesan, Dev Anand, Prem Nazir, Uttam Kumar, Rajendra Kumar, Rajesh Khanna, Kamal Hassan, Mamooty, Chiranjeevi, Mohan Lal, Shah Rukh Khan, Salman… the list of charismatic screen heroes never seems to end, not even after the inclusion of the greatest legend of them all so far, Amitabh Bacchan. Aishwarya Rai's mug adorns not only the hind screen of buses in Southern Karnataka, but even motels in Malibu and bars in Manila.

The frenzy of adulating masses, the culture of fan mail and the collection of memorabilia and pictures of icons of this ilk, speaks of an unfulfilled thirst among the admirers for something far beyond the tangible. For the charismatic, the frenetic awe of the followers becomes an addiction with all the characteristics of withdrawal and dependence.

Writers are also charismatics as we have observed in the case of Harriet Stowe. George Mikes, keen and humorous observer of men and manners, was so awe-inspired by the writings of Arthur Koestler, that as a fellow alien in England, he sought him out. Koestler gave him a decent welcome but remarked that going to see your favorite author was like going to see a turkey because you liked turkey soup. Karnataka's pantheon of Jnanapeeth awardees includes both charismatic and non-charismatic figures, but the writer whose charisma is legendary is T. P. Kailasam, of whom the late Dr. Achar was a great fan.

In Maharashtra, the humorist and essayist Pu La Deshpande, an admirer himself of Kailasam's wit, enjoyed the reputation of being officially acknowledged as the state's darling. This was because of his fearless and uncommonly jocular style of communication. He taught a whole generation to be light-hearted but purposeful. In my family, we would joke that next to our *Kuladaivata* (family deity), we worshipped PuLa-daivata.

Some charismatic artists like Paul Gaugin, Pablo Picasso, and M. F. Hussain have eccentricities that have reinforced public curiosity and bolstered the charisma; sometimes such eccentricities seem deliberately cultivated like the attention seeking behavior of some of our patients.

Such oddities are marked in the world of music too. Edward Elgar often considered the greatest of musician composers in the 20^th^ century was known for his ebullient wit and irreverence. In our own times charismatic Lata Mangeshkar, the Bharat Ratna nightingale guarded her pivotal top position with paranoid alacrity disallowing even siblings to come too close to the top. Gimmickry is resorted to by some musicians to ensure they are better remembered: Bhimsen Joshi and Balamurali Krishna were notorious for such pranks. But to offset such vanities, another well worshipped nightingale, the *saatvik* (virtuous) M. S. Subbalakshmi was rare for the absence of disagreeable quirks.

Rap artist Eminem has reacted to violent childhood abuse in lyrics of hurt and vengeance, as have many modern songwriters. A whole derelict generation woeful, angst ridden and valueless in so many ways, clings to the voices it finds and goes beyond identifying with them.

Remarkable again is the number of musicians across a wide time span who have lives of bereavement or privation in early years, and go on to achieve charismatic success.

## CHARISMA, PHYSICALITY, AND GLAMOUR

The word charisma is probably maximally bandied about today as a synonym for sex appeal. Physical attractiveness and various kinds of athletic prowess are easy gateways to seemingly endless admiration.

Sports icons, models from the worlds of fashion and advertisement, and film stars all have tremendous exposure to public adoration that seems to render them charismatic. It is astonishing how often the beauty is not accompanied by tangible intelligence and even more astounding how little this matters to adoring fans.

What becomes bothersome is the public's losing sight of the idol's limited field of competence. Tabu's opinion about women's issues or Shah Rukh Khan's about nationalism, Vyjayantimala's about religion or Tendulkar's on the purpose of life, belong at best to the realm of interesting trivia. The media too often sets these up as headline material. Where charisma is developed purely on account of glamour or athletic prowess, it often has a short life, stretching commonly for a quarter or half a decade, and rarely through up to three decades. Such is the life of super stars. Their passing has a grand name in physics: supernova.

The latest ‘ism’? ‘Lookism’: An influential academic psychologist, Gordon Patzer has written a new bestseller in ‘Looks: Why they matter more than you ever imagined’.[17] This book seeks to analyze physical attractiveness as a most desirable commodity. To be fair, Patzer does take a sharp look in his work on the darker side of the phenomenon of excessive preoccupation with bodily beauty and the horrific tragedies that have happened as a result, but the vital message is for greater physical attractiveness in this age of fleeting and superficial values.

## CHARISMA, MENTAL HEALTH AND A FEW WORDS OF CAUTION

To begin with ourselves: The cult of charismatic therapists has already been alluded to.

Freud inspired awe and brooked no opposition, thereby losing many an adroit follower but building for his reputation an unconquerable castle. Adler was far more accommodating though considerably less charismatic; Jung even as he differed from Freud in his ideas, and eventually broke with him, built an aura around his persona akin to that of Freud, his first mentor. [18]

Charisma has been considered by some to be essentially pathological, and not uncommonly it obviously is. However, to call somebody charismatic is always a compliment, never pejorative. The public identity is constructed many a time in the absence of a secure and satisfactory private self.[2] The risk of narcissism developing can never be over-emphasized.[19] There is also a close relationship to paranoid tendencies and hypomanic behavior, given the charismatic's heightened sense of self-importance.

Parental deprivation as well as bouts of personal angst or depression, are fore-runners of the condition and the emotion of despondency may be well masked but might continue to run along the life lines of the charismatic. Charismatic appeal has more than one meaning. It is a powerful aesthetic attraction to the public at one level. It also is a cry for attention and help, artfully disguised or transcended. The public responds to the appeal with feeling sometimes being reduced to mindless automatons if the hold of the charismatic is firm. There are also issues here that resemble the features of transference that we are familiar with.

Charismatics seeking union with their subjects/fans/adulators often end up having intimacies of varying kinds. Charismatic leaders from various walks are famous for their libidinous lives. Even the charismatic therapist is at great risk for dalliances that could be his/her undoing.

Mental health specialists are often aware that large numbers of persons needing their care end up seeking the help of charismatic healers of various hues: from the well meaning to outrageous charlatans. The reasons at one level are obvious: no stigma of seeing a shrink, wider fellow feeling with other followers and not at all uncommonly, a much less expensive regimen to follow. It would be pompous and presumptuous to dismiss out of hand that at least a few do benefit in mental health in such encounters. To my mind, the main problem is that these charismatic healing sessions are never open to evaluation or scrutiny. Hence, arises the need to be cautious if not wary.

The lesson of the Jonestown massacre is just three decades old and needs our careful attention.[20] The truly altruistic and vastly talented Jim Jones was the charismatic father figure who gave salvation to thousands in his commune that espoused admirable values of equality, responsibility and non-violence. Respected politicians patronized him and the world acknowledged his extraordinary humanitarian talents. As his strength grew, he seems to have descended into delusionally seeing himself as a messiah and savior of his followers in a world doomed to nuclear destruction. From Indiana to California to Guyana he moved his devoted flock, all of them living largely peaceful lives. Allegations of coercion and abuse against this commune brought forth a government sponsored enquiry that caused rumblings in the Temple commune leading to murders and mass suicide that spelt the ruination of a well begun and unprecedented project of human brotherhood.

I conclude with the acidic ruminations of Philip Rieff:

*“Is it not likely that since all action is now decided from the outside in, that a spray-on charisma can will soon be invented? We shall have the recognition factors sorted out, and so by purchase smartly in the economy size, spray ourselves into something extraordinary. This is simply to say what Soren Kierkegaard said more than a century ago, that all inwardness is lost.”*[3]

In my younger days one rarely saw posters of a political leader; maybe a few odd ones at election time. Today the local satrap, councilor, MLA and/or MP beam at me from hoardings crowded with their faces along with those of their cronies. If we are lucky, a few of those faces smile. The hoardings are at every corner and cross road. They boast of their birthdays, they wish us for festival days, Sundays, Mondays….. They wish basically that we take notice and that they remain a visible presence thrust upon us. Charisma, no longer divine grace, is a parody of itself; a purchasable commodity when there is the right patronage.

Friends, thank you for your patient listening.
